# Supplementary material for: aPEAR: an R package for autonomous visualization of pathway enrichment networks
Source: Bioinformatics. 2023 Nov 3;39(11):btad672. doi: 10.1093/bioinformatics/btad672 (PMC10641035; doi:10.1093/bioinformatics/btad672)
Supplement: btad672_Supplementary_Data [file btad672_supplementary_data.pdf]

Supplementary information

Supplementary Text 1. The detailed description of the input format for the *aPEAR* package.

For more detailed and up-to-date information, please check the vignette of the aPEAR package.

The most important function exported by the *aPEAR* package is `enrichmentNetwork()`. This function accepts the enrichment results as a *data.frame* object. When using *gprofiler2* (Kolberg, Raudvere, Kuzmin, Vilo, & Peterson, 2020) or *clusterProfiler* (Wu et al., 2021), the *aPEAR* package will handle all necessary actions when provided with the *data.frame* of the results from these methods, for example:

```
> # Perform enrichment analysis
> enrich <- clusterProfiler::gseGO(geneList, OrgDb = org.Hs.eg.db, ont = "CC")
>
> # Generate enrichment network using aPEAR
> aPEAR::enrichmentNetwork(enrich@result)
```

When using gprofiler2, do not forget to set `evcodes = TRUE` in order to obtain the gene list for each pathway.
In case the enrichment analysis is carried out differently, the package can still be utilised to create the enrichment network. The data should be formatted as a *data.frame* object that comprises the following columns:

- ***Description*** - this column contains the titles of the pathways and will be used to generate the cluster names.
- ***pathwayGenes*** - this column contains the list of genes in the pathway. The user may choose to use the complete list of genes in the pathway or only the core enrichment genes.
- A column that will be used to colour the nodes (we recommend using p-values or NES). The user has to specify which column to use with `enrichmentNetwork(..., colorBy = "columnName")`.
- A column that will be used to choose the size of the node (we recommend using the number of genes in the pathway). The user has to specify which column to use with `enrichmentNetwork(..., nodeSize = "columnName")`.

The ***Description*** and ***pathwayGenes*** columns for custom inputs are **required**. The columns for node size and colour have to be specified, so the user can choose them freely. For example:

```
> # This is an example of correctly formatted enrichment result
> # (can contain other columns as well):
> enrich[ 1:5, ]

##                                Description                        pathwayGenes       NES size
## 1: Abc-Family Proteins Mediated Transport  11160/27248/51009/6400/10956/... -2.865165  103
## 2:        Acetyl-Coa Biosynthetic Process       1738/1737/5162/38/23417/... -2.442777   13
## 3:           Acetyl-Coa Metabolic Process        1738/1737/5162/38/3158/... -2.655216   24
## 4:          Activation Of Immune Response      929/7100/1191/6850/10392/...  2.201129  300
## 5:          Acyl-Coa Biosynthetic Process 1738/1737/5162/9524/109703458/... -3.026606   38
```

```
> # Generate enrichment network using aPEAR
> aPEAR::enrichmentNetwork(enrich, nodeSize = "size", colorBy = "NES")
```

Note, that it does not matter which gene identifiers are used as long as they are the same between all pathways. For more information on the available methods and plotting parameters, please check `?aPEAR.methods` and `?aPEAR.theme`.

Supplementary Text 2. Usage of *aPEAR* to obtain clusters of redundant pathways.

We also provide a simple way to obtain the pathway clustering results. This can be done with `findPathClusters()`. The function accepts a *data.frame* in the same format as `enrichmentNetwork()` and returns a list of clusters (*data.frame*) and the similarity matrix (*matrix*) which can later be used to create the enrichment network by `plotPathClusters()`. These two methods are internally called by `enrichmentNetwork()` and generate the same result.

```
> # Get clustering result using aPEAR
> data <- aPEAR::findPathClusters(enrich@result)
> data$clusters[ 1:3 ]

##                                       ID                                  Cluster
## 1:                           CMG complex   DNA replication preinitiation complex
## 2:                 DNA packaging complex                               nucleosome
## 3: DNA replication preinitiation complex   DNA replication preinitiation complex

> # Create the enrichment network visualisation using default parameters
> aPEAR::plotPathClusters(enrich@result, data$sim, data$clusters)
```

Supplementary Text 3. The evaluation of clustering quality.

To determine which similarity metric and clustering algorithm produces the best results, we performed clustering evaluation using multiple cluster quality indexes that look at various aspects of the data. Specifically, we used the Dunn index, the Silhouette index, and the Davies-Bouldin index. The Dunn index measures the separation between clusters and the compactness of each cluster (Dunn, 1974). The Silhouette index indicates how well each data point fits into its assigned cluster, based on the average distance between the point and all other points in its cluster as well as the average distance between the point and all points in the nearest neighbouring cluster (Rousseeuw, 1987). The Davies-Bouldin index evaluates the average similarity between each cluster and its most similar cluster (Davies & Bouldin, 1979).

The clusters obtained by spectral clustering algorithm received the worst estimates by all quality indexes which significantly differed from the metrics obtained by the hierarchical and the Markov clustering algorithms ($p\text{-value} < 0.01$, Wilcoxon signed-rank test) (Supplementary Fig. 5a,c). The Dunn index estimates were similar for the hierarchical and the Markov clustering algorithms, however, the Silhouette and the Davies-Bouldin indexes showed

significant preference for the Markov clustering as compared to the hierarchical clustering (p-value < 0.01, Wilcoxon signed-rank test) (Supplementary Fig. 5a,c). All cluster quality indexes showed that the similarity metric did not have an impact on the cluster quality (p-value > 0.01, Wilcoxon signed-rank test) (Supplementary Fig. 5b,c). Based on this evaluation, we decided to set the default similarity metric to the Jaccard similarity metric as it is most frequently used by other similar tools (Merico, Isserlin, Stueker, Emili, & Bader, 2010; Yu, 2022), and to set the default clustering algorithm to the Markov clustering algorithm as it showed the best clustering quality. The spectral clustering did not prove to be applicable to the gene-set cluster analysis but the implementation was retained for experimental purposes, however, it should be used with care.

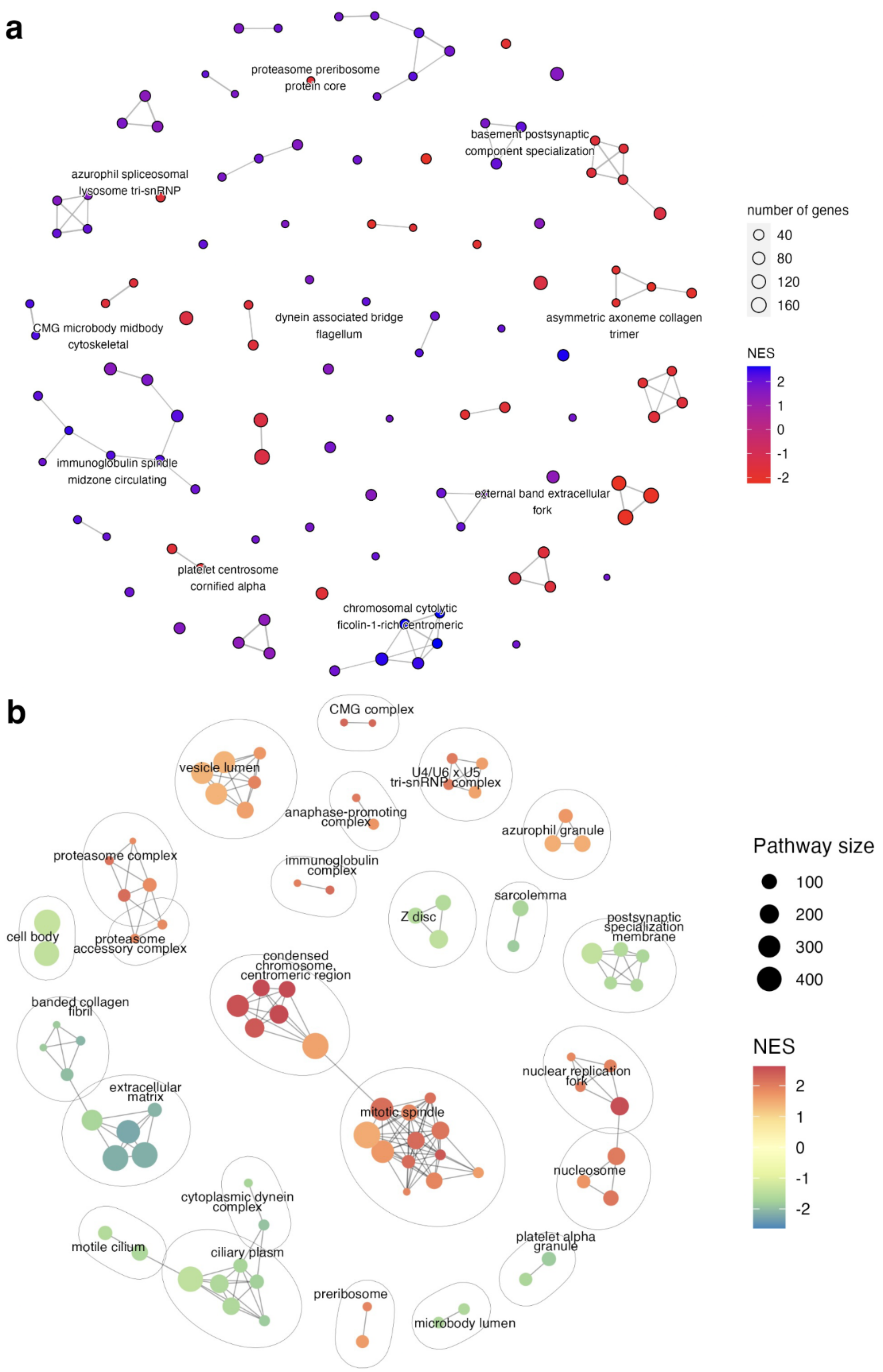

Supplementary Figure 1. Enrichment networks of the *clusterProfiler* results, generated using *emapplot* (a) and *aPEAR* (b). The GSEA was performed using the example gene list from the R package *DOSE*.

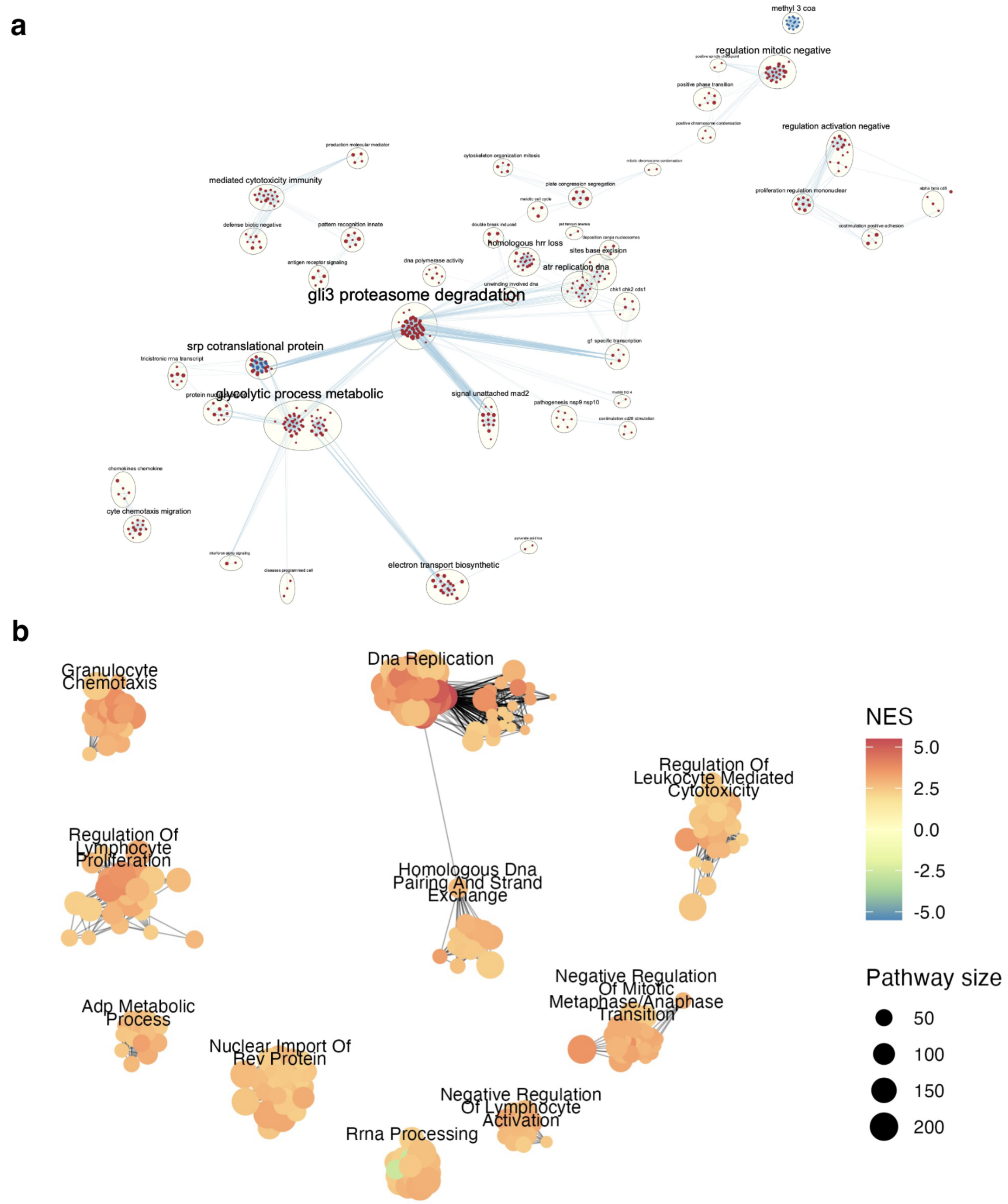

Supplementary Figure 2. Enrichment networks of the GSEA software results, generated using *Cytoscape* (a) and *aPEAR* (b). The GSEA was performed using the example gene list from the R package *DOSE*.

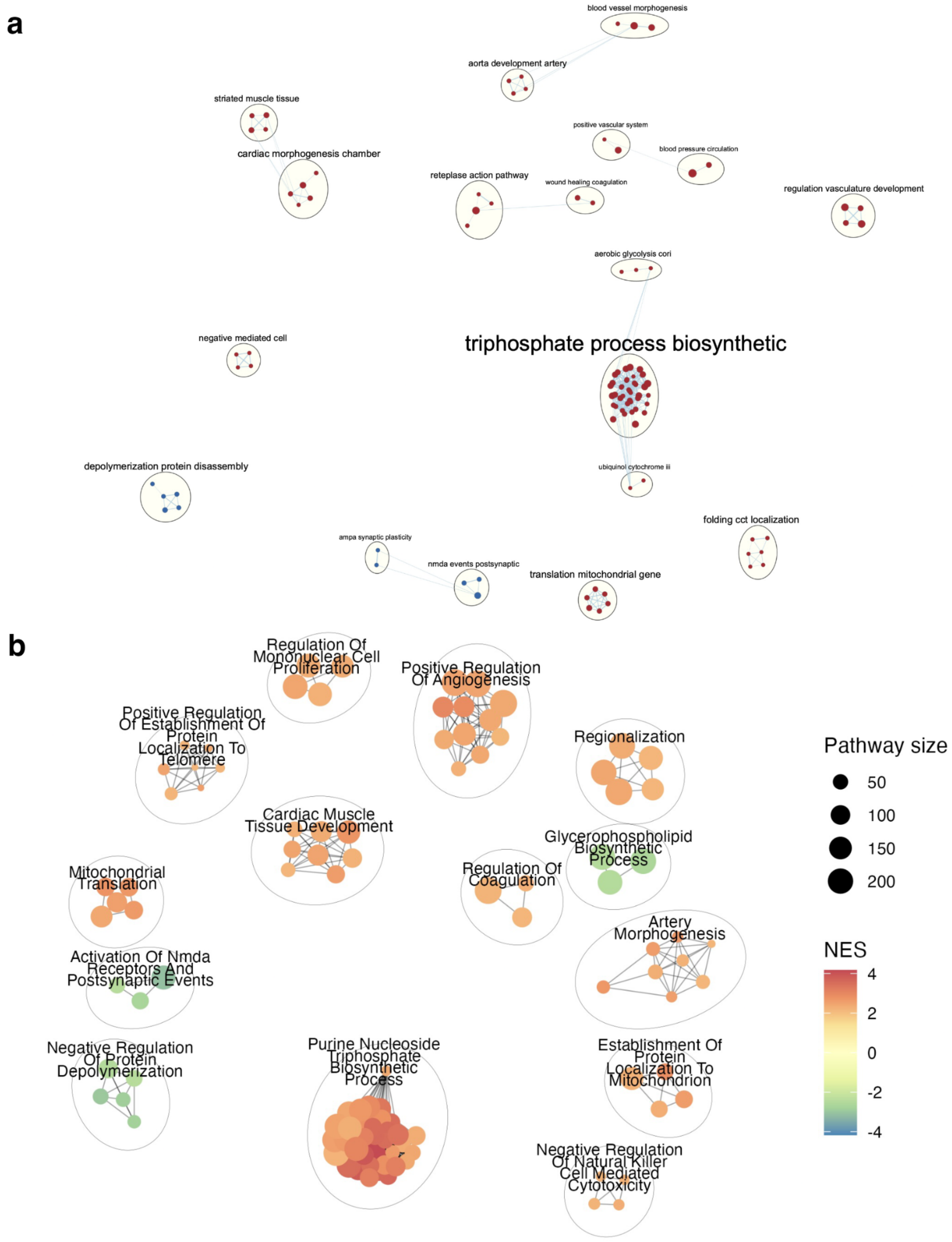

Supplementary Figure 3. Enrichment networks of the GSEA software results, generated using *Cytoscape* (a) and *aPEAR* (b). The GSEA was performed using a gene list from an internal analysis using single cell RNA sequencing data from human samples.

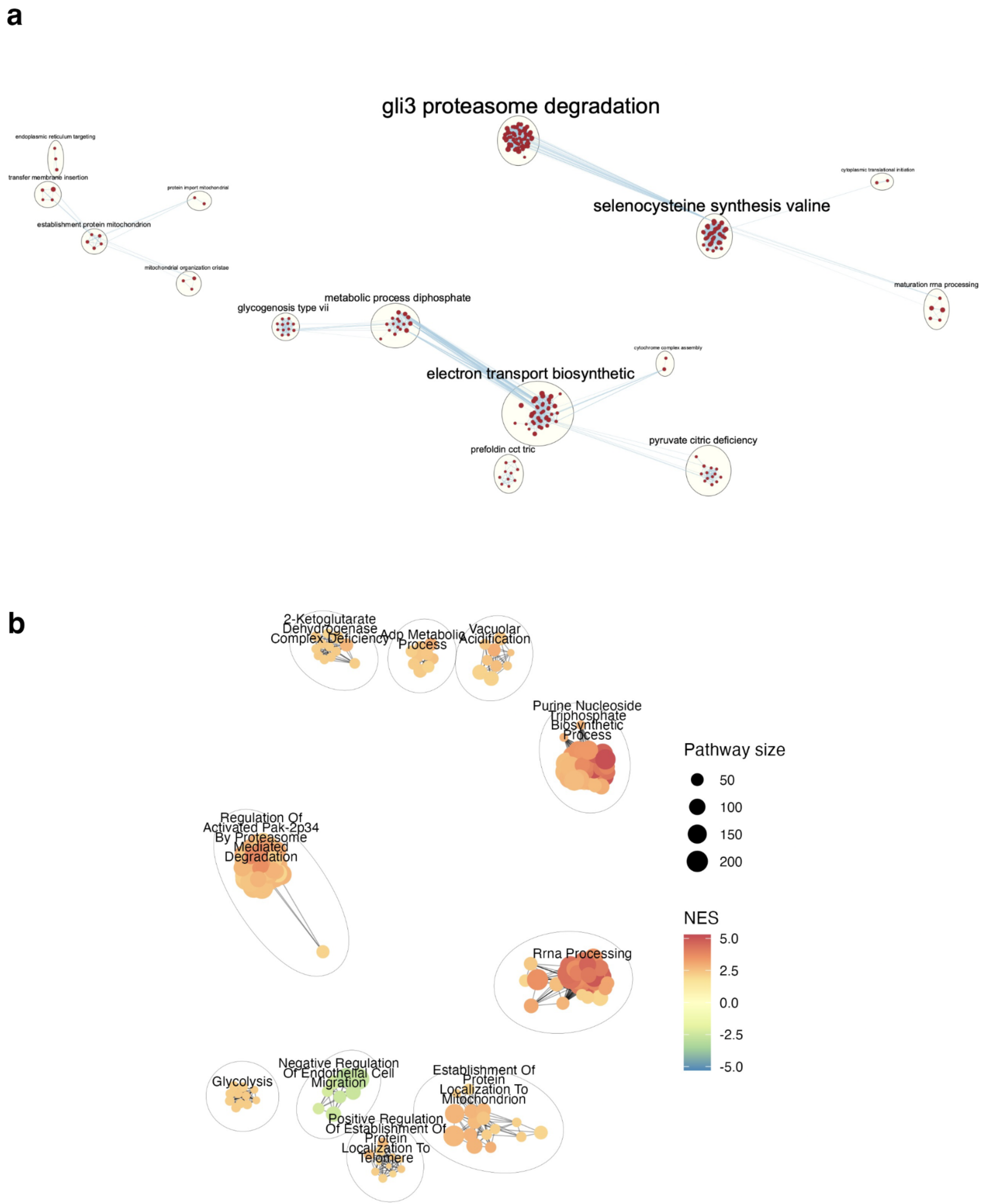

Supplementary Figure 4. Enrichment networks of the GSEA software results, generated using *Cytoscape* (a) and *aPEAR* (b). The GSEA was performed using a gene list from an internal analysis using single cell RNA sequencing data from human samples.

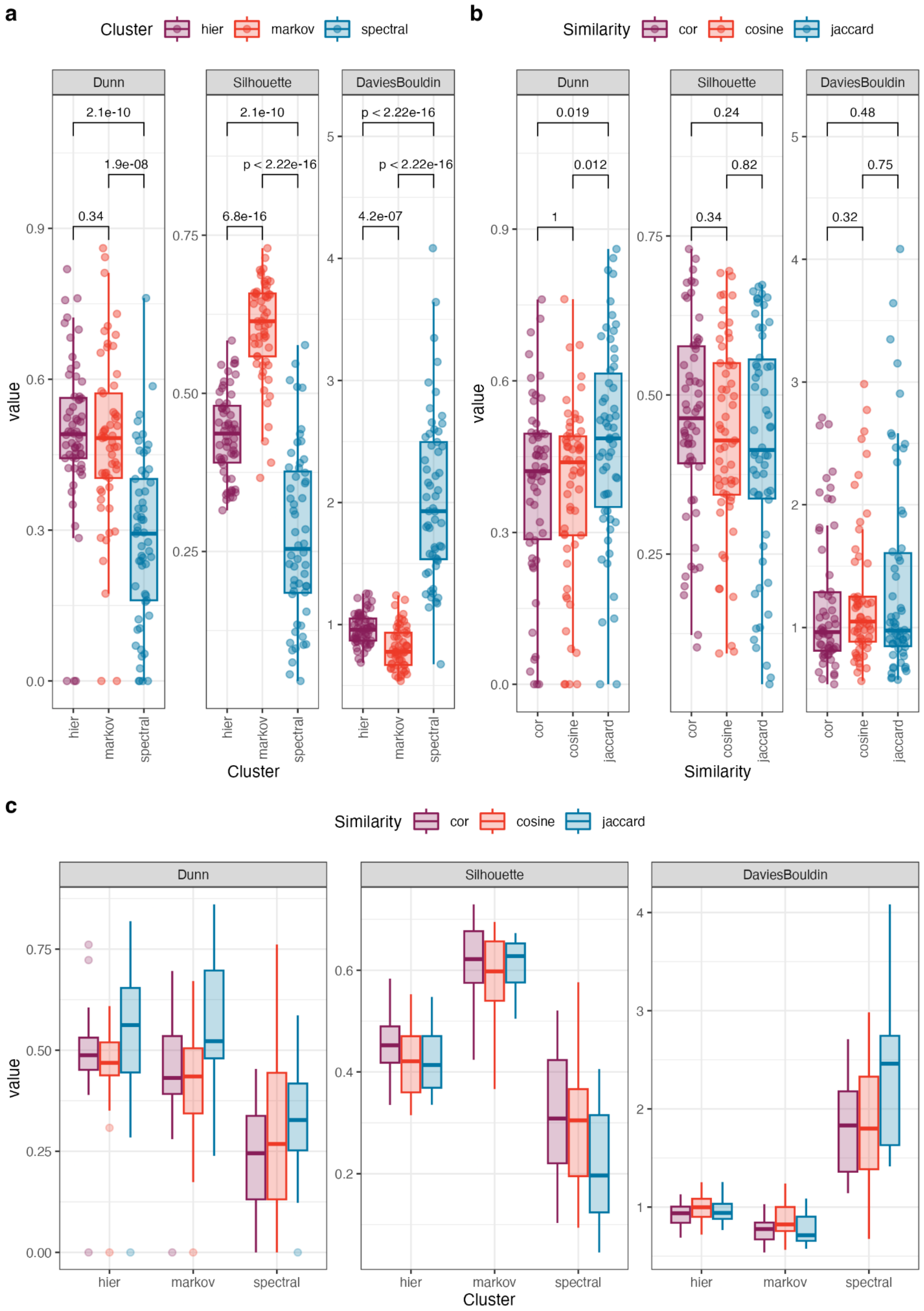

Supplementary Figure 5. Evaluation of cluster quality using various similarity and clustering metrics. Cluster quality dependency on clustering method (a) and similarity metric (b). The p-values obtained using the Wilcoxon signed-rank test. The figure (c) describes the cluster quality dependency on the clustering method split by the similarity metric.

Supplementary Table 1. Datasets used for clustering quality evaluation. 10 different real-world datasets were used to perform gene set enrichment using two methods: *gseGO* from R package *clusterProfiler* (Wu et al., 2021) and the GSEA software (Mootha et al., 2003; Subramanian et al., 2005). The enrichment results (top 1000 most significant pathways) can be found in the *aPEAR* publication GitHub repository.

No	Enrichment method	Data type	Condition
1.	*GSEA*	RNA-Seq	Alzheimer's disease
2.	*clusterProfiler*		
3.	*GSEA*	RNA-Seq	SARS-CoV-2 (nasal swab)
4.	*clusterProfiler*		
5.	*GSEA*	EPIC methylation	ovarian cancer
6.	*clusterProfiler*		
7.	*GSEA*	EPIC methylation	persons who gave birth to newborns with FGR
8.	*clusterProfiler*		
9.	*GSEA*	EPIC methylation	Alzheimer's disease
10.	*clusterProfiler*		
11.	*GSEA*	EPIC methylation	mild Alzheimer's disease
12.	*clusterProfiler*		
13.	*GSEA*	WGBS	severe SARS-CoV-2
14.	*clusterProfiler*		
15.	*GSEA*	EPIC methylation	persons who gave birth to newborns with autism spectrum disorder
16.	*clusterProfiler*		
17.	*GSEA*	RNA-Seq	late-stage Parkinson's disease
18.	*clusterProfiler*		
19.	*GSEA*	deep RNAseq	Parkinson's disease
20.	*clusterProfiler*		

Supplementary Table 2. Clustering quality metrics when using various clustering and similarity metrics. Obtained using the top 100 significant pathways from the datasets described in Supplementary Table 1.

Dataset	Cluster	Similarity	Dunn	Silhouette	DaviesBouldin
1	markov	jaccard	0.23861	0.60606	0.79508
1	hier	jaccard	0.40959	0.44477	0.86839
1	spectral	jaccard	0	0.26236	1.61759
1	markov	cosine	0.41301	0.59782	0.75348
1	hier	cosine	0.37603	0.51779	0.85383
1	spectral	cosine	0	0.19516	2.41451
1	markov	cor	0.42954	0.57805	0.75033
1	hier	cor	0.38926	0.51875	0.86575
1	spectral	cor	0.05387	0.39577	1.22259
2	markov	jaccard	0.6635	0.65121	0.59279
2	hier	jaccard	0.56549	0.41744	0.92327
2	spectral	jaccard	0.37688	0.18737	2.34632
2	markov	cosine	0.5091	0.60969	0.70699
2	hier	cosine	0.44295	0.45271	0.83126
2	spectral	cosine	0.26803	0.24377	2.24216
2	markov	cor	0.48783	0.65272	0.62222
2	hier	cor	0.49179	0.47626	0.80844
2	spectral	cor	0.32056	0.33427	2.13894
3	markov	jaccard	0.37606	0.6521	0.77625
3	hier	jaccard	0.28424	0.46867	0.92932
3	spectral	jaccard	0.37467	0.34957	1.41494
3	markov	cosine	0.34306	0.69495	0.791
3	hier	cosine	0.48927	0.46569	0.98048
3	spectral	cosine	0.23861	0.36826	1.24758
3	markov	cor	0.28467	0.67702	0.63603
3	hier	cor	0.49577	0.47321	0.92944
3	spectral	cor	0.23869	0.43584	1.14038
4	markov	jaccard	0.52219	0.54446	0.71245
4	hier	jaccard	0.75718	0.40504	0.86847
4	spectral	jaccard	0.34263	0.11515	2.9056

4	markov	cosine	0.48289	0.61397	0.6681
4	hier	cosine	0.46457	0.41829	0.90512
4	spectral	cosine	0.06213	0.18261	2.16207
4	markov	cor	0.57633	0.6559	0.57983
4	hier	cor	0.4628	0.42335	0.85872
4	spectral	cor	0.24525	0.19943	2.27021
5	markov	jaccard	0.52629	0.62784	0.90372
5	hier	jaccard	0.59544	0.51265	0.84139
5	spectral	jaccard	0.12248	0.28061	1.92639
5	markov	cosine	0.29438	0.58975	0.98917
5	hier	cosine	0.46908	0.42855	1.01535
5	spectral	cosine	0.48827	0.36048	1.52205
5	markov	cor	0.49789	0.67938	0.76904
5	hier	cor	0.4831	0.44047	0.97001
5	spectral	cor	0.421	0.42437	1.63337
6	markov	jaccard	0.7299	0.65328	0.64509
6	hier	jaccard	0.62826	0.34303	0.97042
6	spectral	jaccard	0.32608	0.05574	3.64232
6	markov	cosine	0.53443	0.55015	0.8846
6	hier	cosine	0.60896	0.36478	1.02807
6	spectral	cosine	0.18838	0.09375	2.98372
6	markov	cor	0.57196	0.5876	0.82818
6	hier	cor	0.60547	0.40283	0.99836
6	spectral	cor	0.41093	0.10329	2.70963
7	markov	jaccard	0.45001	0.61459	0.9331
7	hier	jaccard	0.50947	0.54504	0.88233
7	spectral	jaccard	0.17282	0.13452	2.46614
7	markov	cosine	0.29713	0.68674	0.9232
7	hier	cosine	0.49631	0.49814	0.88642
7	spectral	cosine	0.4777	0.24843	1.92879
7	markov	cor	0.41317	0.7141	0.82725
7	hier	cor	0.56027	0.54695	0.80696
7	spectral	cor	0	0.25967	1.79166
8	markov	jaccard	0.84268	0.6416	0.58693

8	hier	jaccard	0.71178	0.45014	0.97973
8	spectral	jaccard	0.12994	0.10249	3.34794
8	markov	cosine	0.50042	0.65799	0.82203
8	hier	cosine	0.56195	0.50813	0.96952
8	spectral	cosine	0.46984	0.19539	2.53648
8	markov	cor	0.49589	0.66083	0.77838
8	hier	cor	0.72285	0.52989	0.88966
8	spectral	cor	0.22961	0.18537	2.64665
9	markov	jaccard	0.47477	0.50454	1.08605
9	hier	jaccard	0.55849	0.33547	1.15861
9	spectral	jaccard	0.32044	0.13277	2.56321
9	markov	cosine	0.34427	0.53347	1.20226
9	hier	cosine	0.46854	0.44189	1.08652
9	spectral	cosine	0.41842	0.35774	1.53555
9	markov	cor	0.414	0.55838	0.93785
9	hier	cor	0.4526	0.44345	1.01919
9	spectral	cor	0.29878	0.22869	2.0443
10	markov	jaccard	0.86062	0.60547	0.67671
10	hier	jaccard	0.81887	0.34703	1.04115
10	spectral	jaccard	0.51651	0.15485	2.4953
10	markov	cosine	0.42091	0.39045	1.05177
10	hier	cosine	0.47307	0.33934	1.04501
10	spectral	cosine	0.76148	0.5763	0.67498
10	markov	cor	0.46475	0.42401	0.97202
10	hier	cor	0.76091	0.39719	0.98471
10	spectral	cor	0.1604	0.21463	2.21778
11	markov	jaccard	0.50734	0.67031	1.00263
11	hier	jaccard	0.42332	0.44891	1.13631
11	spectral	jaccard	0.52972	0.23806	1.95272
11	markov	cosine	0.46571	0.69183	0.76679
11	hier	cosine	0.44476	0.3958	1.2173
11	spectral	cosine	0.39468	0.32456	1.63722
11	markov	cor	0.43139	0.69713	0.85612
11	hier	cor	0.4511	0.47908	1.09558

11	spectral	cor	0.2929	0.23018	1.83174
12	hier	jaccard	0.68264	0.40973	1.02991
12	spectral	jaccard	0.58612	0.19653	2.45999
12	hier	cosine	0.3507	0.33164	1.25203
12	spectral	cosine	0.29515	0.28504	1.85932
12	hier	cor	0.49478	0.38716	1.08105
12	spectral	cor	0.45381	0.31444	1.8129
13	markov	jaccard	0.48748	0.59323	1.04356
13	hier	jaccard	0.45007	0.39865	0.95092
13	spectral	jaccard	0.45896	0.40584	1.64412
13	markov	cosine	0.43511	0.65671	1.15911
13	hier	cosine	0.52333	0.36476	1.06308
13	spectral	cosine	0.27627	0.38293	1.57875
13	markov	cor	0.27984	0.59014	0.94866
13	hier	cor	0.5155	0.43232	0.95523
13	spectral	cor	0.40172	0.44331	1.42728
14	markov	jaccard	0.4076	0.52711	0.90023
14	hier	jaccard	0	0.3425	1.25433
14	markov	cosine	0	0.36638	1.23888
14	hier	cosine	0	0.34328	1.19179
14	markov	cor	0	0.48436	1.02836
14	hier	cor	0	0.39153	1.12954
15	markov	jaccard	0.48478	0.65917	0.6688
15	hier	jaccard	0.42981	0.54755	0.86747
15	spectral	jaccard	0.25825	0.37461	1.57243
15	markov	cosine	0.38534	0.65662	0.63388
15	hier	cosine	0.52594	0.48425	0.88447
15	spectral	cosine	0		1.18501
15	markov	cor	0.38009	0.72949	0.60987
15	hier	cor	0.56565	0.50228	0.77945
15	spectral	cor	0.025	0.42262	1.25565
16	markov	jaccard	0.81146	0.64545	0.62651
16	hier	jaccard	0.62081	0.37608	0.96969
16	spectral	jaccard	0.45826	0.07458	3.15218

16	markov	cosine	0.66612	0.54656	0.77476
16	hier	cosine	0.42265	0.31512	1.08316
16	spectral	cosine	0.4906	0.10553	2.7723
16	markov	cor	0.61023	0.5214	0.7755
16	hier	cor	0.41574	0.33522	1.07019
16	spectral	cor	0.24896	0.12284	2.65623
17	markov	jaccard	0.48483	0.54416	0.82846
17	hier	jaccard	0.48955	0.34784	1.09449
17	spectral	jaccard	0.24658	0.38211	1.54395
17	markov	cosine	0.46297	0.49542	0.98161
17	hier	cosine	0.30804	0.34553	1.19259
17	spectral	cosine	0.16987	0.54673	1.21856
17	markov	cor	0.40352	0.57238	0.82113
17	hier	cor	0.46478	0.45088	0.94292
17	spectral	cor	0.04874	0.52076	1.27399
18	markov	jaccard	0.68826	0.67304	0.57419
18	hier	jaccard	0.64436	0.53479	0.76654
18	spectral	jaccard	0.3061	0.20473	2.58228
18	markov	cosine	0.5873	0.63353	0.56437
18	hier	cosine	0.53535	0.55286	0.71937
18	spectral	cosine	0.10484	0.33363	1.79958
18	markov	cor	0.69589	0.67661	0.53762
18	hier	cor	0.52122	0.58346	0.68757
18	spectral	cor	0.10195	0.30864	2.05184
19	markov	jaccard	0.53056	0.66292	0.66523
19	hier	jaccard	0.54468	0.47598	0.90583
19	spectral	jaccard	0.34256	0.38996	1.47986
19	markov	cosine	0.17374	0.44669	1.01181
19	hier	cosine	0.44287	0.42369	0.95953
19	spectral	cosine	0.15754	0.50891	1.17335
19	markov	cor	0.36075	0.5782	0.73762
19	hier	cor	0.42173	0.4854	0.84102
19	spectral	cor	0.2324	0.51015	1.2916
20	markov	jaccard	0.70568	0.55863	0.67938

20	hier	jaccard	0.69872	0.40465	0.89001
20	spectral	jaccard	0.32739	0.0453	4.08377
20	markov	cosine	0.67079	0.58796	0.75775
20	hier	cosine	0.51805	0.41542	0.91892
20	spectral	cosine	0.07012	0.09667	2.59817
20	markov	cor	0.65258	0.62181	0.70445
20	hier	cor	0.45168	0.45389	0.83463
20	spectral	cor	0.35472	0.22621	2.10047

Supplementary Methods

Generating the input for *aPEAR*, *emapplot* and *Enrichment Map* visualisations

To generate the enrichment networks using the *emapplot* (Yu, 2022) and the *Enrichment Map* (Merico et al., 2010), we performed gene set enrichment analysis using the example gene list from R package *DOSE* (Yu, Wang, Yan, & He, 2015) and two internal datasets. These datasets originate from 10X single cell RNA sequencing of human samples. The data were preprocessed with *cellranger* (Zheng et al., 2017) using hg38 human reference genome and R package *Seurat* (Butler, Hoffman, Smibert, Papalexi, & Satija, 2018; Hao et al., 2021; Satija, Farrell, Gennert, Schier, & Regev, 2015; Stuart et al., 2019) following the best practices (Luecken & Theis, 2019). Differential gene expression analysis compared gene expression between two conditions using *limma* (Ritchie et al., 2015) and the resulting gene rankings were used to perform gene set enrichment analysis. The gene rankings are available for download on the GitHub repository (https://github.com/ievaKer/aPEAR-publication).

As *emapplot* works best with *gseaResult* (or *enrichResult*) objects as input, we performed the analysis using the *gseGO* method from *clusterProfiler* (Wu et al., 2021) with default parameters and cellular component gene ontology. For the *Enrichment Map* we performed the analysis using the GSEA software (Mootha et al., 2003; Subramanian et al., 2005) with 1000 permutations, classic scoring scheme and *meandiv* normalisation method; the pathways were filtered to contain a minimum of 10 and a maximum of 200 genes. We used the GMT file provided by the Bader Lab, Toronto (Human GO:BP All Pathways).

Cytoscape and *Enrichment Map*

The Cytoscape software and its plugin *Enrichment Map* (Merico et al., 2010) can be used to generate an enrichment network. However, this process is difficult to automate, requires manual rerun when the enrichment results change, and the visualisation may still require some improvements by hand. We used the R package *RCy3* (Gustavsen, Pai, Isserlin, Demchak, & Pico, 2019) to generate the enrichment network using the Cytoscape software and its plugin *Enrichment Map* directly from an R script. It requires that the Cytoscape software is running in the background, also, we had to include a sleeping time period in order to make sure that the results in the Cytoscape software are properly updated. We used the enrichment results obtained from the GSEA software and ran the *enrichmentmap mastermap* command with q-value cutoff 0.05, Jaccard similarity metric with the similarity cutoff 0.5 and did not perform filtering by expression. Then, we removed the edges and nodes with few connections and annotated the graph with *autoannotate* (Kucera, Isserlin, Arkhangorodsky, & Bader, 2016).

emapplot

The *emapplot* visualisation was created using the *clusterProfiler (Wu et al., 2021)* enrichment output. First, pairwise-termism was calculated using the *pairwise_termsim* method from the R package *enrichplot* (Yu, 2022), with the *showCategory* parameter set to the size of the complete enrichment dataset. Then, the *emapplot* function was used to generate the enrichment network with node colour set to *NES* and node label set to *group*.

Cluster quality evaluation

We used 10 different real-life datasets (Supplementary Table 1) and performed enrichment analysis using *clusterProfiler* (Wu et al., 2021), as well as the GSEA software (Mootha et al., 2003; Subramanian et al., 2005). We used the GMT file provided by the Bader Lab, Toronto (Human GO:BP All Pathways). This resulted in 20 different gene sets which were then used to evaluate the clustering quality with all the implemented similarity metrics (Jaccard index, cosine similarity score, and correlation) and clustering algorithms (hierarchical, spectral, and Markov clustering). In total, we measured the quality of 180 gene set clusters obtained from *aPEAR* (Supplementary Table 2).

References

Bader Lab, University Of Toronto. "Human GO:BP All Pathways", http://download.baderlab.org/EM_Genesets/January_01_2023/Human/entrezgene/Human_GOBP_AllPathways_no_GO_iea_January_01_2023_entrezgene.gmt. Accessed Jan 30. (2023, January 1). .

Butler, A., Hoffman, P., Smibert, P., Papalexi, E., & Satija, R. (2018). Integrating single-cell transcriptomic data across different conditions, technologies, and species. *Nature Biotechnology*, *36*(5), 411–420.

Davies, D. L., & Bouldin, D. W. (1979). A cluster separation measure. *IEEE transactions on pattern analysis and machine intelligence*, *1*(2), 224–227.

Dunn, J. C. (1974). Well-Separated Clusters and Optimal Fuzzy Partitions. *Journal of Cybernetics*, *4*(1), 95–104.

Gustavsen, J. A., Pai, S., Isserlin, R., Demchak, B., & Pico, A. R. (2019). RCy3: Network biology using Cytoscape from within R. [version 3; peer review: 3 approved]. *F1000Research*, *8*, 1774.

Hao, Y., Hao, S., Andersen-Nissen, E., Mauck, W. M., Zheng, S., Butler, A., Lee, M. J., et al. (2021). Integrated analysis of multimodal single-cell data. *Cell*, *184*(13), 3573-3587.

Kolberg, L., Raudvere, U., Kuzmin, I., Vilo, J., & Peterson, H. (2020). gprofiler2 -- an R package for gene list functional enrichment analysis and namespace conversion toolset g:Profiler. *F1000Research*, *9*, 709.

Kucera, M., Isserlin, R., Arkhangorodsky, A., & Bader, G. D. (2016). AutoAnnotate: A Cytoscape app for summarizing networks with semantic annotations. [version 1; peer review: 2 approved]. *F1000Research*, *5*, 1717.

Luecken, M. D., & Theis, F. J. (2019). Current best practices in single-cell RNA-seq analysis: a tutorial. *Molecular Systems Biology*, *15*(6), e8746.

Merico, D., Isserlin, R., Stueker, O., Emili, A., & Bader, G. D. (2010). Enrichment map: a network-based method for gene-set enrichment visualization and interpretation. *Plos One*, *5*(11), e13984.

Mootha, V. K., Lindgren, C. M., Eriksson, K.-F., Subramanian, A., Sihag, S., Lehar, J., Puigserver, P., et al. (2003). PGC-1alpha-responsive genes involved in oxidative phosphorylation are coordinately downregulated in human diabetes. *Nature Genetics*, *34*(3), 267–273.

Ritchie, M. E., Phipson, B., Wu, D., Hu, Y., Law, C. W., Shi, W., & Smyth, G. K. (2015). limma powers differential expression analyses for RNA-sequencing and microarray studies. *Nucleic Acids Research*, *43*(7), e47.

Rousseeuw, P. J. (1987). Silhouettes: A graphical aid to the interpretation and validation of cluster analysis. *Journal of Computational and Applied Mathematics*, *20*, 53–65.

Satija, R., Farrell, J. A., Gennert, D., Schier, A. F., & Regev, A. (2015). Spatial reconstruction of single-cell gene expression data. *Nature Biotechnology*, *33*(5), 495–502.

Stuart, T., Butler, A., Hoffman, P., Hafemeister, C., Papalexi, E., Mauck, W. M., Hao, Y., et al. (2019). Comprehensive Integration of Single-Cell Data. *Cell*, *177*(7), 1888-1902.e21.

Subramanian, A., Tamayo, P., Mootha, V. K., Mukherjee, S., Ebert, B. L., Gillette, M. A., Paulovich, A., et al. (2005). Gene set enrichment analysis: a knowledge-based approach for interpreting genome-wide expression profiles. *Proceedings of the National Academy of Sciences of the United States of America*, *102*(43), 15545–15550.

Wu, T., Hu, E., Xu, S., Chen, M., Guo, P., Dai, Z., Feng, T., et al. (2021). clusterProfiler 4.0: A universal enrichment tool for interpreting omics data. *Innovation (Cambridge (Mass.))*, *2*(3), 100141.

Yu, G., Wang, L.-G., Yan, G.-R., & He, Q.-Y. (2015). DOSE: an R/Bioconductor package for disease ontology semantic and enrichment analysis. *Bioinformatics*, *31*(4), 608–609.

Yu, G. (2022). enrichplot: Visualization of Functional Enrichment Result.

Zheng, G. X. Y., Terry, J. M., Belgrader, P., Ryvkin, P., Bent, Z. W., Wilson, R., Ziraldo, S. B., et al. (2017). Massively parallel digital transcriptional profiling of single cells. *Nature Communications*, *8*, 14049.
